# Gene expression analysis reveals diabetes-related gene signatures

**DOI:** 10.1186/s40246-024-00582-z

**Published:** 2024-02-08

**Authors:** M. I. Farrim, A. Gomes, D. Milenkovic, R. Menezes

**Affiliations:** 1 CBIOS, Universidade Lusófona’s Research Center for Biosciences & Health Technologies, Universidade Lusófona, Lisbon, Portugal; 2 Universidad de Alcalá, Escuela de Doctorado, Madrid, Spain; 3 Department of Nutrition, University of California Davis, Davis, USA

**Keywords:** Diabetes, Integrative bioinformatics, lncRNAs, miRNAs, Genomics, Transdifferentiation

## Abstract

**Background:**

Diabetes is a spectrum of metabolic diseases affecting millions of people worldwide. The loss of pancreatic β-cell mass by either autoimmune destruction or apoptosis, in type 1-diabetes (T1D) and type 2-diabetes (T2D), respectively, represents a pathophysiological process leading to insulin deficiency. Therefore, therapeutic strategies focusing on restoring β-cell mass and β-cell insulin secretory capacity may impact disease management. This study took advantage of powerful integrative bioinformatic tools to scrutinize publicly available diabetes-associated gene expression data to unveil novel potential molecular targets associated with β-cell dysfunction.

**Methods:**

A comprehensive literature search for human studies on gene expression alterations in the pancreas associated with T1D and T2D was performed. A total of 6 studies were selected for data extraction and for bioinformatic analysis. Pathway enrichment analyses of differentially expressed genes (DEGs) were conducted, together with protein–protein interaction networks and the identification of potential transcription factors (TFs). For noncoding differentially expressed RNAs, microRNAs (miRNAs) and long noncoding RNAs (lncRNAs), which exert regulatory activities associated with diabetes, identifying target genes and pathways regulated by these RNAs is fundamental for establishing a robust regulatory network.

**Results:**

Comparisons of DEGs among the 6 studies showed 59 genes in common among 4 or more studies. Besides alterations in mRNA, it was possible to identify differentially expressed miRNA and lncRNA. Among the top transcription factors (TFs), HIPK2, KLF5, STAT1 and STAT3 emerged as potential regulators of the altered gene expression. Integrated analysis of protein-coding genes, miRNAs, and lncRNAs pointed out several pathways involved in metabolism, cell signaling, the immune system, cell adhesion, and interactions. Interestingly, the GABAergic synapse pathway emerged as the only common pathway to all datasets.

**Conclusions:**

This study demonstrated the power of bioinformatics tools in scrutinizing publicly available gene expression data, thereby revealing potential therapeutic targets like the GABAergic synapse pathway, which holds promise in modulating α-cells transdifferentiation into β-cells.

**Supplementary Information:**

The online version contains supplementary material available at 10.1186/s40246-024-00582-z.

## Introduction

Diabetes is among the most common chronic diseases worldwide and continues to increase as changing lifestyles lead to increased obesity. According to the 2021 report from the International Diabetes Federation (IDF), the global diabetes prevalence in 20–79-year-olds in 2021 was estimated to be 10.5% (536.6 million people), rising to 12.2% (783.2 million) in 2045 [1]. Despite efforts to advance knowledge about the disease and to develop novel therapeutic strategies, diabetes still represents a significant social and economic burden partially due to the lack of effective therapeutics to prevent β-cell loss and dysfunction. People with type 1 (T1D) and type 2 (T2D) diabetes represent two different phenotypes concerning age at onset, diabetes duration, and lifetime glycemic load [2]. However, loss of β-cell mass and function with subsequent insulin deficiency represent important events in disease pathogenesis. Therefore, restoring functional pancreatic β-cells is the biggest challenge in treating diabetes [3].

In recent years, the focus of research on the mechanisms of diabetes has shifted to the molecular level, including the study of disturbed epigenetic modifications and abnormal noncoding RNA (ncRNA) expression [4, 5]. The cell's biological system is incredibly complex and includes components at different levels of gene expression, such as DNA, messenger RNA (mRNA), ncRNAs such as microRNAs (miRNAs) and long noncoding RNAs (lncRNAs), and proteins. Each of these levels can be modulated by different elements causing alterations in cell function [6]. MiRNAs and lncRNAs are single-stranded RNA molecules of approximately 22 and more than 200 nucleotides in length, respectively [7]. They are involved in the regulation of different processes, including the cell cycle and cellular differentiation, with possible roles in multiple physiological and pathological processes, such as cell interactions, immune responses, and other cellular functions, via post-translational modifications. Recent studies have linked the role of some miRNAs and lncRNAs with diabetes pathogenesis in cell functions associated with insulin function, β-cell activity, and glucose metabolism [8–10]. Moreover, studies suggest that ncRNAs may serve as modulators and diagnostic markers of diabetic cardiovascular disease [11].

Owing to the multifactorial and complex processes underlying diabetes pathophysiology, traditional bench science, where only one level of gene expression regulation is evaluated, provides an incomplete picture of the mechanisms involved [12]. Genomics strategies are put in place to overcome this limitation by integrating two or more types of genomic data to create a global network of biological interactions [6]. Data-driven research based on integrative genomics has the potential to unravel disease mechanisms and has become indispensable for the deep understanding of the physiological processes and complex mechanisms underlying disease onset and progression of heterogeneous diseases such as diabetes [9, 12, 13]. Large-scale studies of data compiled from different genomic studies can alleviate the biases of individual studies and unveil possible disease biomarkers and molecular targets amenable to therapeutic intervention [6].

Integrated genomics studies are still scarce in the context of diabetes [14], mainly in T1D. Concerning T2D, several studies using individual genomics approaches have been reported; however, these tend to be underpowered due to small sample sizes and lack of consensus on the protocols used, yielding inconsistent findings [9]. Notwithstanding, further integrative studies are still needed to compile data from complementary omics to add more pieces to the puzzle of the molecular mechanisms underlying diabetes pathogenesis [9, 15].

To fill this knowledge gap, we used publicly available data on gene expression and regulatory noncoding RNAs and integrated these data using computational biology to identify molecular targets and networks associated with diabetes. The integrated analysis of protein-coding genes, miRNAs, and lncRNAs identified several pathways involved in the regulation of cellular functions, such as metabolism, cell signaling, the immune system, cell adhesion, and interactions. The GABAergic synapse pathway was identified as the common pathway to all analyses. The regenerative capacity of γ-aminobutyric acid (GABA) has been studied in conditions of β-cell depletion, where GABA seems to contribute to the transdifferentiation of α-cells into β-cells. Our study reinforces the importance of this pathway as a potential target for improving insulin production in diabetes.

## Methods

### Strategy for literature research and data extraction

**Fig. 1:**
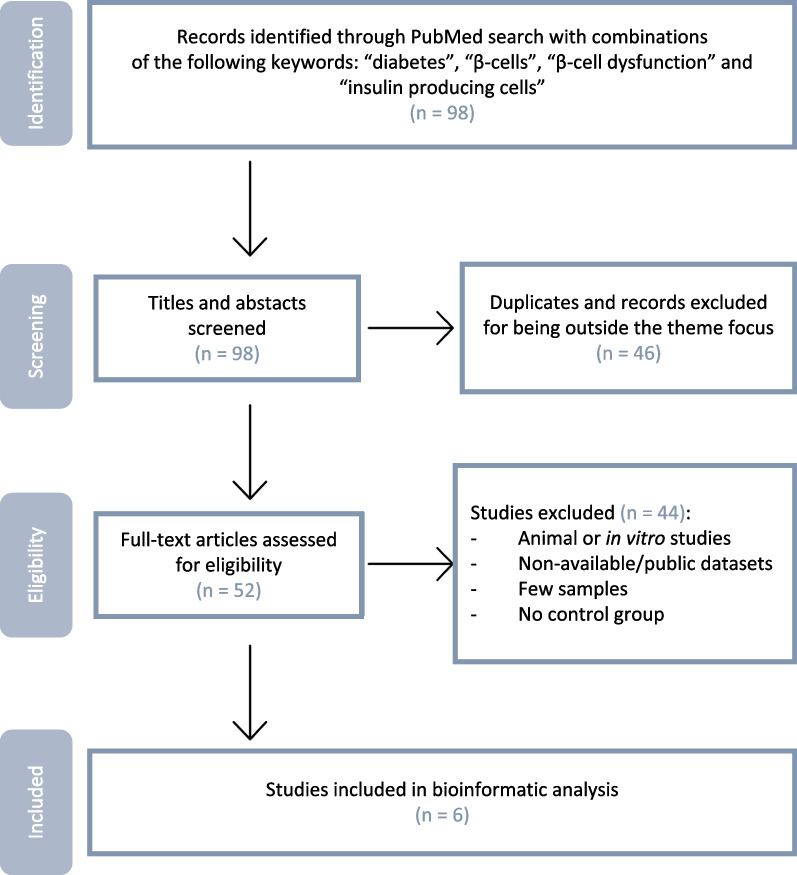
Flowchart of the studies selected for bioinformatic analysis

Literature on diabetes-associated gene expression alterations in PubMed was conducted from May to June 2022 for combinations of the keywords “diabetes,” “β-cells,” “β-cell dysfunction” and “insulin-producing cells.” The inclusion criteria were as follows: (1) studies evaluating pancreatic islet samples; (2) studies reporting donors without diabetes (ND) and donors with diabetes (DD); and (3) studies providing publicly available annotation data (Fig. 1). Only human studies with publicly available datasets and that considered a control group (non-diabetic individuals) were used. Six studies, denoted as GSE164416 [16], GSE25724 [17], GSE20966 [18], GSE76894 [19], GSE86473 [20], and GSE124742 [21] (Table 1), were selected for data collection. For 4 of the studies (GSE25724, GSE164416, GSE20966 and GSE76894), the data were analyzed with GEO2R tool, available at the Gene Expression Omnibus (GEO) repository. The samples were divided into DD vs ND groups, and the following settings were applied: Benjamini & Hochberg was used for *p* value adjustment with a significance cutoff of 0.05. No log transformation or forced normalization was applied. For the 2 remaining studies (GSE86473 and GSE124742), the differently expressed genes (DEGs) were obtained from the supplementary material available with the original article. Both studies used methods previously validated and applied R and python scripts to obtain DEGs.

**Table 1:** Characterization of the studies selected for bioinformatic analysis

Study	Dataset	Human pancreatic islets samples		Sample isolation method	Protocol design
		Total n°	Type		
Doi: 10.1038/s42255-021-00420-9	GSE164416	57	18 ND, 39 T2D	Laser capture microdissection	SMART-seq Illumina Hiseq 2500 or 500
Doi: 10.1074/jbc.M110.200295	GSE25724	13	7 ND, 6 T2D	Collagenase digestion followed by density gradient purification	HG- U133A Affymetrix chips
Doi: 10.1371/journal.pone.0011499	GSE20966	20	10 ND, 10 T2D	Laser capture microdissection	DNA-Chip Analyzer
Doi: 10.1007/s00125-017-4500-3	GSE76894	103	84 ND, 19 T2D	Enzymatic digestion or laser capture microdissection	Affymetrix Human Genome U133 Plus 2.0 Array
Doi: 10.1101/gr.212720.116	GSE86473	8	5 ND, 3 T2D	Cultured and isolated via C1 integrated fluidic circuit	SMART-seq2 Illumina NextSeq500
Doi: 10.1016/j.cmet.2020.04.005	GSE124742	6	3 ND, 3 T1D	Patch-seq and FACS	SMART-seq2 Illumina NextSeq500 or Novaseq platform

*ND* individuals without diabetes, *T2D* individuals with type 2 diabetes, *T1D* individuals with type 1 diabetes, *FACS* fluorescence-activating cell sorting

Table 1 contains the details of the selected studies with the respective accession numbers after the selection process. The full table of differentially expressed genes identified for each study analyzed can be found in Additional file 1.

### Bioinformatic analysis

To account for changes within datasets, each dataset was first analyzed individually. Only differentially expressed genes (*p* value < 0.05) were extracted and subjected to bioinformatic analyses. The ShinyGO [22] (http://bioinformatics.sdstate.edu/go/) online tool was used for the identification of the different types of RNA (mRNA, lncRNA, miRNA) present in the studies.

### Individual genomics-layer network construction

#### mRNA network

Potential transcription factors (TFs) were identified with the bioinformatic tool Enrichr [23] (https://amp.pharm.mssm.edu/Enrichr/) as a platform to interrogate two transcription factor databases, TRRUST [24] and TRANSFAC [25]. The ClustVis [26] (https://biit.cs.ut.ee/clustvis/) tool was used to generate the fold-change heatmap.

#### Protein network

For the protein‒protein interaction (PPI) network, STRING [27] (Protein‒Protein Interaction Networks Functional Enrichment Analysis) software v11.5 (https://string-db.org/) was used to identify the interactions between nodes that had a confidence score of 0.7. The proteins with no connections were removed from the network.

#### lncRNA and miRNA networks

LncRNAs and their respective targets were identified using the online tool LncRRIsearch [28] (http://rtools.cbrc.jp/LncRRIsearch/index.cgi?t4=&hist=&em=em15) considering the energy threshold of -20 kcal/mol. miRNA and the respective targets were identified using the online tools miRBase [29] (https://www.mirbase.org/index.shtml) and MIENTURNET [30] (http://userver.bio.uniroma1.it/apps/mienturnet/). Only the targets with *p* value < 0.05 were considered for further analysis.

#### Pathway analysis

Pathway enrichment analyses were conducted using the bioinformatic tool GeneTrail [31] v.3.2 (https://genetrail2.bioinf.uni-sb.de/) to access the Kyoto Encyclopedia of Genes and Genomes (KEGG) [32] and WikiPathways databases. The following settings were applied: over-representation analysis with a two-sided null hypothesis and no adjustment of the *p* value. Only the pathways with a *p* value < 0.05 were analyzed, and the pathways associated with other diseases (such as cancer, infections, and others) were removed. R studio software 2023.03.0 + 386 with the package “ggplot2” was used to generate the bubble plots.

#### Multilayer integration and network analysis

InteractiVenn [33] (http://www.interactivenn.net/) was used to discover common elements between different datasets. In Cytoscape software [34] (version 3.10; https://cytoscape.org/), interactions between mRNA-TFs, miRNA-targets, lncRNA-targets, and protein‒protein were visualized. The KEGG Mapper database (https://www.genome.jp/kegg/mapper/color.html) tool was used to visualize and construct the GABAergic synapse pathway. Genes associated with diabetes were retrieved from the Genome-Wide Association Studies (GWAS) Catalog (https://www.ebi.ac.uk/gwas/).

## Results

### Study selection and analysis

A comprehensive search on PubMed, conducted in May–June 2022, identified 98 publications. The literature search was conducted with combinations of the following keywords: diabetes, β-cells, β-cell dysfunction, and insulin-producing cells (Fig. 1). After the removal of duplicates and records outside the intended scope, this number dropped to 52 manuscripts, which were further screened and assessed for eligibility. A detailed full-text analysis of these 52 studies led to the exclusion of 44 studies reporting animal and in vitro studies as well as manuscripts with non-available data in public databases or that did not consider a control group (individuals without diabetes). A total of 6 studies, denoted as GSE164416 [16], GSE25724 [17], GSE20966 [18], GSE76894 [19], GSE86473 [20], and GSE124742 [21] (Table 1), were selected for data extraction and considered eligible for bioinformatic analysis. They included only reports with significant changes in gene expression of pancreatic islets from human donors, analyzed by microarray, RNA-seq, and single-cell sequencing. The study selection process for bioinformatic analysis is depicted in Fig. 1**.** The study population comprises 5 studies including T2D and ND donors (GSE164416 [16], GSE25724 [17], GSE20966 [18], GSE76894 [19], GSE86473 [20]) and 1 study including T1D and ND donors (GSE124742 [21]). Males and females were found in equal numbers in all studies. The average age of donors was 55 years old, with donors in the T1D study being younger (an average age of 32 years old). The body mass index (BMI) of the donors ranged between 23 and 34 kg/m^2^.

### Integrated analysis of differentially expressed genes and pathways

**Fig. 2:**
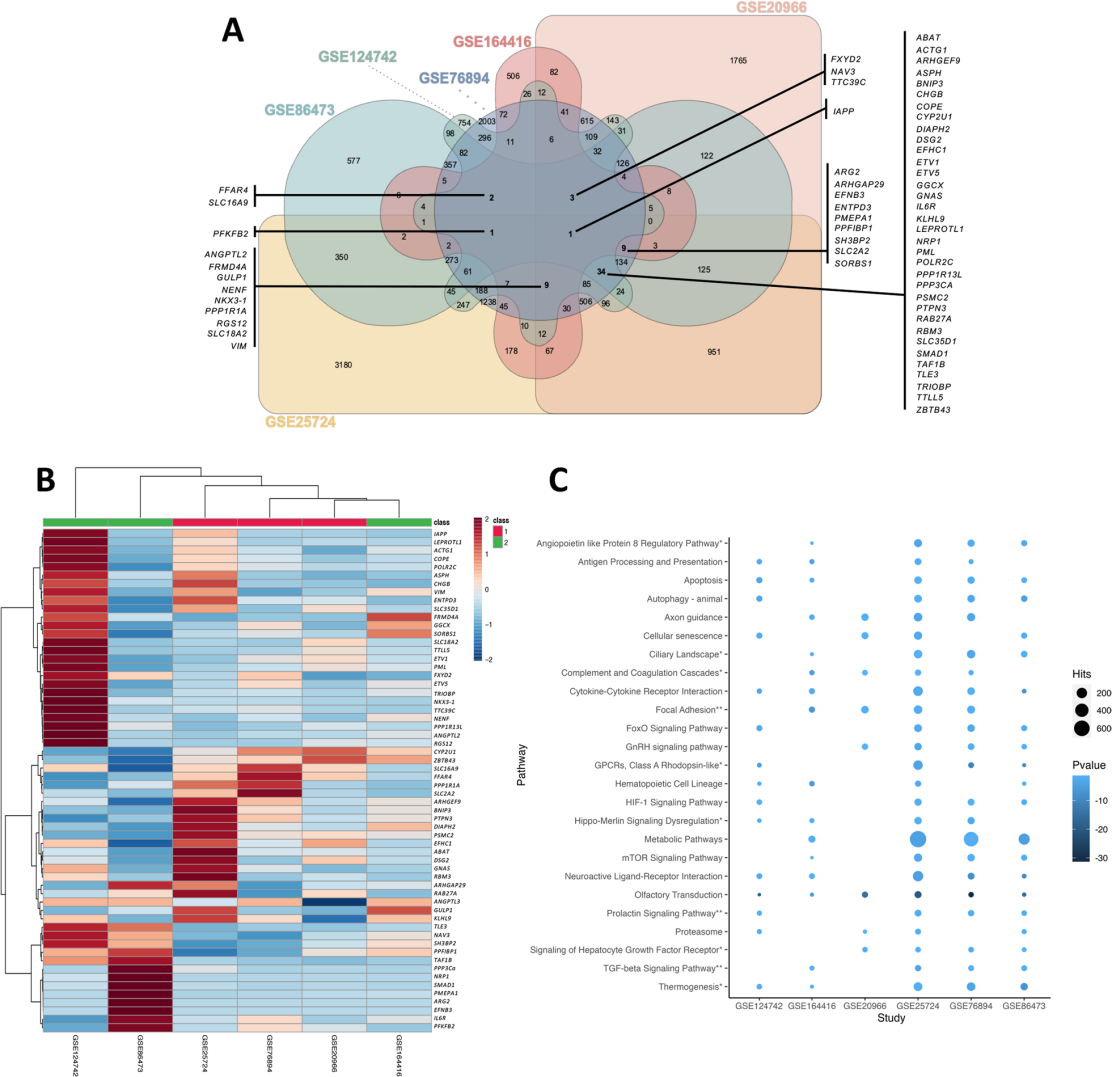
Differentially expressed genes (DEGs) in the selected studies. **A** Venn diagram of DEGs from each study and their intersections. The genes presented are common to at least 4 selected studies. **B** Heatmap of the DEGs common to at least 4 of the studies. Class 1 and Class 2 correspond to microarrays and RNA-seq/single-cell studies, respectively. Red and blue colors represent up- and downregulated genes, respectively. **C** Bubble plot of the functional enriched pathways of the mRNA genes for each study. The size and color of the dots represent the number of hits and the range of the pathway’s *p* values, respectively. KEGG pathways are represented with no asterisk, WikiPathways with *, and pathways common to both databases are represented with **

The number of DEGs between ND/DD samples in each study varied from 868 to 7083. Among these, differentially expressed mRNAs, lncRNAs, and miRNAs were analyzed for each study (Table 2). Globally, ~ 96.9% of the genes corresponded to protein-coding genes, 2.6% to lncRNAs, and nearly 0.5% to miRNAs. These data indicate that not only protein-coding genes but also regulatory RNAs such as lncRNAs and miRNAs are altered in DD samples. Comparisons of DEGs among the 6 studies showed 59 genes in common among 4 or more studies (Fig. 2A). The fold change of these genes was evaluated in the different studies, showing that generally ~ 59.3% of the DEGs were upregulated, while ~ 40.7% were downregulated. Notwithstanding, there were dissimilarities in the pattern of regulation between different studies, as observed by the different colors in the heatmap (Fig. 2B).

**Table 2:** Different types of RNA present in each study

Data set	mRNA		lncRNA		miRNA		Total
	*N*	%	*N*	*%*	*N*	%	
GSE164416	868	90.70	89	9.30	0	0.00	957
GSE25724	7083	97.95	94	1.30	54	0.75	7231
GSE20966	4240	95.41	204	4.59	0	0.00	4444
GSE76894	5626	96.92	132	2.27	47	0.81	5805
GSE86473	2299	99.27	15	0.65	2	0.09	2316
GSE124742	1999	96.85	63	3.05	2	0.10	2064

*mRNA* messenger RNA, *lncRNA* long noncoding RNA, *miRNA* microRNA

The differentially expressed protein-coding genes from each study were subjected to pathway enrichment analyses using GeneTrail, and the pathways common to at least 4 studies are represented in Fig. 2C. The studies analyzed by microarrays had a higher number of altered pathways and several hits. These pathways are involved in cell signaling of HIF-1, FoxO, GnRH, TGF-β, the immune system, including cytokine interactions and antigen presentation, cell adhesion and interactions, metabolism, nervous system-like axon guidance, and other cellular functions, such as proteasome and apoptosis. Olfactory transduction appeared to be a common pathway in all the studies. Although not common to all the studies, metabolic pathways had the highest number of hits.

### Protein‒protein interaction analysis

**Fig. 3:**
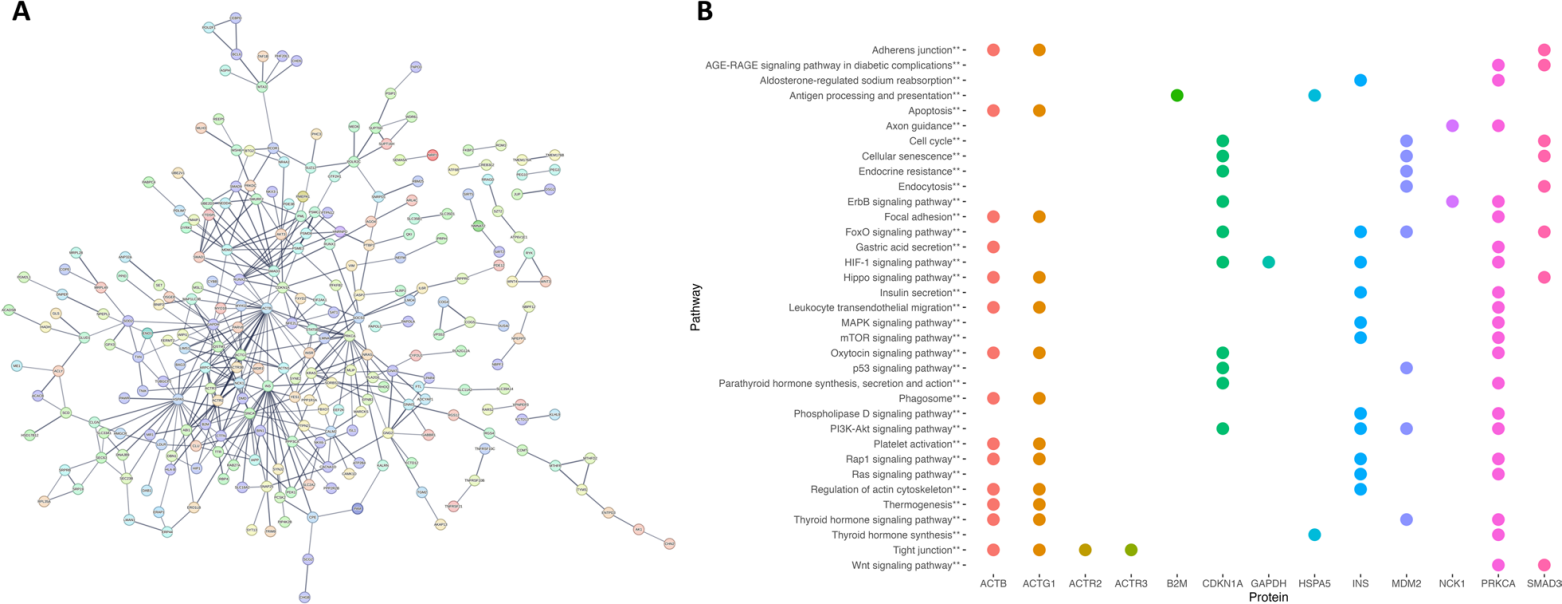
Functional interaction of common differentially expressed proteins. **A** Network of protein‒protein interactions with a confidence score of 0.7. **B** Heatmap of the pathways associated with the proteins with the highest number of interactions. Each protein is represented by a different color. KEGG pathways are represented with no asterisk, WikiPathways with *, and pathways common to both databases are represented with **

Potential interactions among proteins encoded by the genes identified as differentially expressed in diabetes were explored using the STRING database. The analysis revealed a network of interactions between different proteins that form nodes in the network (Fig. 3A). We next selected the proteins with the highest number of interactions potentially playing important roles in the regulation of different cellular functions. The number of interactions reached 29 for ACTB (β Actin), 19 for HSPA5 (Heat Shock Protein Family A), 18 for INS (Insulin), 15 for GAPDH (Glyceraldehyde-3-Phosphate Dehydrogenase), and 14 for ACTG1 (Actin γ-1) and PKCA (Protein Kinase C α). Interestingly, pathway enrichment analysis of the top 13 proteins (ACTB, HSPA5, INS, GAPDH, ACTG1, PKCA, SMAD3, ACTR2, CDKN1A, MDM2, ACTR3, B2M and NCK1) with more interactions, conducted in GeneTrail, revealed that these proteins are involved in insulin secretion, regulation of actin cytoskeleton, tight junction, FoxO and HIF-1 signaling pathways, among others (Fig. 3B).

### Transcription factors regulating the expression of diabetes-modulated genes

**Fig. 4:**
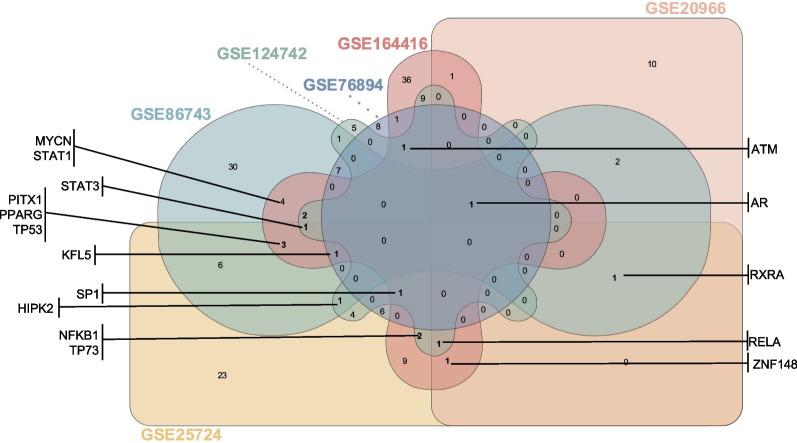
Differentially expressed transcription factors (TFs) and targets. Venn diagram of the differentially expressed TFs for the different studies. Only the TFs highlighted are common to at least 3 of the publications in the analysis

Using the Enrichr platform, the next step was to take the gathered data on differentially expressed genes to search for potential TFs whose regulatory function could be modulated by diabetes. We identified 178 transcription factors, from which 16 potential TFs were found to be common to 3 or more studies: ataxia telangiectasia mutated (ATM), androgen receptor (AR), homeodomain interacting protein kinase 2 (HIPK2), Kruppel-like factor 5 (KFL5), MYCN proto-oncogene BHLH transcription factor (MYCN), nuclear factor kappa B subunit 1 (NFKB1), paired like homeodomain 1 (PITX1), peroxisome proliferator activated receptor gamma (PPARG), RELA proto-oncogene NF-KB subunit (RELA), retinoid X receptor α (RXRA), Sp1 transcription factor (SP1), signal transducer and activator of transcription 1 (STAT1), signal transducer and activator of transcription 3 (STAT3), tumor protein p53 (TP53), tumor protein p73 (TP73) and zinc finger protein 148 (ZNF148) (Fig. 4**)**. The network construction showed the interconnectivity between the main TFs and their extensive network with more than 4000 targets (Additional file 2: Fig. S1). Therefore, the activity of these TFs could be affected by diabetes and be responsible for the regulation of identified differentially expressed genes.

### LncRNAs and miRNAs expression altered in diabetes

The gene expression data allowed the identification of altered expression patterns of noncoding RNAs, particularly miRNAs and lncRNAs, associated with diabetes.

**Fig. 5:**
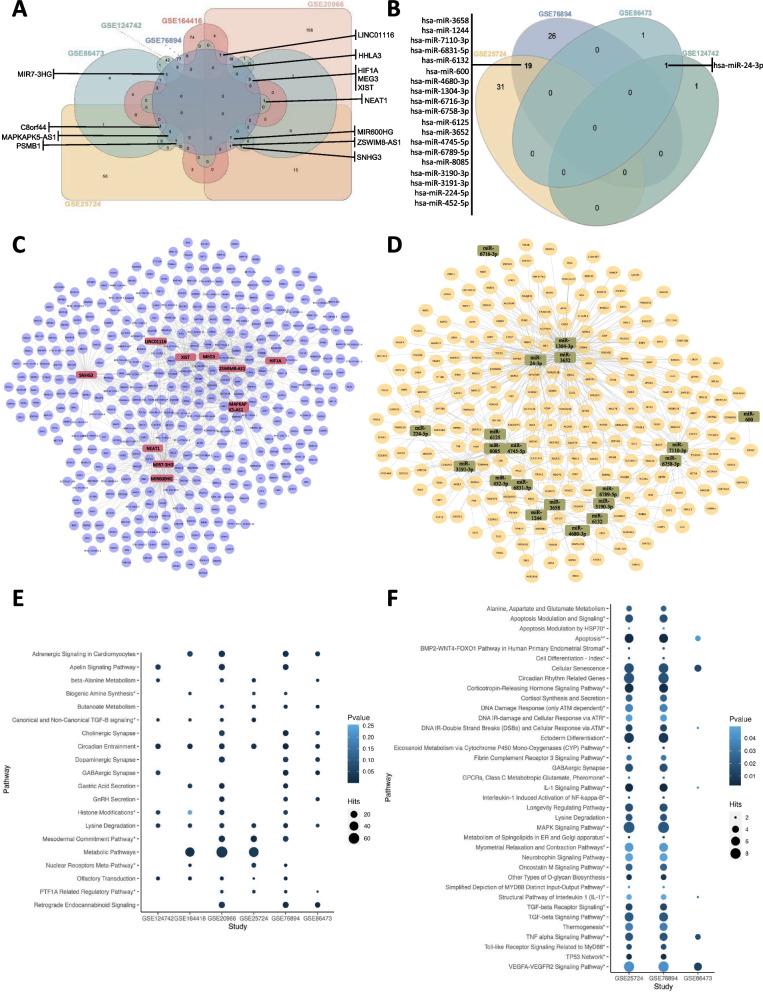
Differentially expressed long noncoding RNA (lncRNA) and microRNA (miRNA) networks and functional pathways. **A**, **B** Venn diagram of the differentially expressed lncRNAs and miRNAs, respectively. The names of lncRNAs common to at least 3 of the studies are shown. **C**, **D** Interaction network of common differentially expressed lncRNAs and miRNAs, respectively. The RNAs correspond to the nodes and their respective targets on the edges. **E**, **F** Bubble plot of the functional enriched pathways of lncRNA and miRNA targets for each study, respectively. KEGG pathways are represented with no asterisk and WikiPathways with *. The size and color of the dots represent the number of hits and the range of the pathway’s *p* value, respectively

Changes were observed in the expression of 597 lncRNAs, of which 13 were common to at least 3 studies (Fig. 5A). The lncRRIsearch database was used to search for the target genes of the identified lncRNAs, and ~ 960 target genes were identified for the 13 common lncRNAs. The network of interactions among the identified lncRNAs and their target genes is presented in Fig. 5C. Identification of lncRNA-associated cellular functions was performed using the respective target genes to reveal pathways from GeneTrail. Metabolic pathways, retrograde endocannabinoid signaling, and circadian entrainment were a few of the main pathways identified in these analyses (Fig. 5E).

Four studies reported changes in the expression of 105 miRNAs associated with diabetes, of which 20 were common between 2 studies (Fig. 5B). The target genes of these miRNAs were identified using MIENTURNET, which revealed 539 target genes. These data were used to construct a network of miRNAs and their targets (Fig. 5D). Functional enrichment analysis was performed using GeneTrail, which allowed the identification of apoptosis, MAPK, and TGF-β/TGF-β receptor signaling as cellular pathways significantly affected by miRNAs common to at least 2 studies (Fig. 5F).

These results support the notion that diabetes is associated with altered expression of specific noncoding RNAs, such as lncRNAs and miRNAs, that can affect many genes in different pathways.

### Integrated functional analysis

**Fig. 6:**
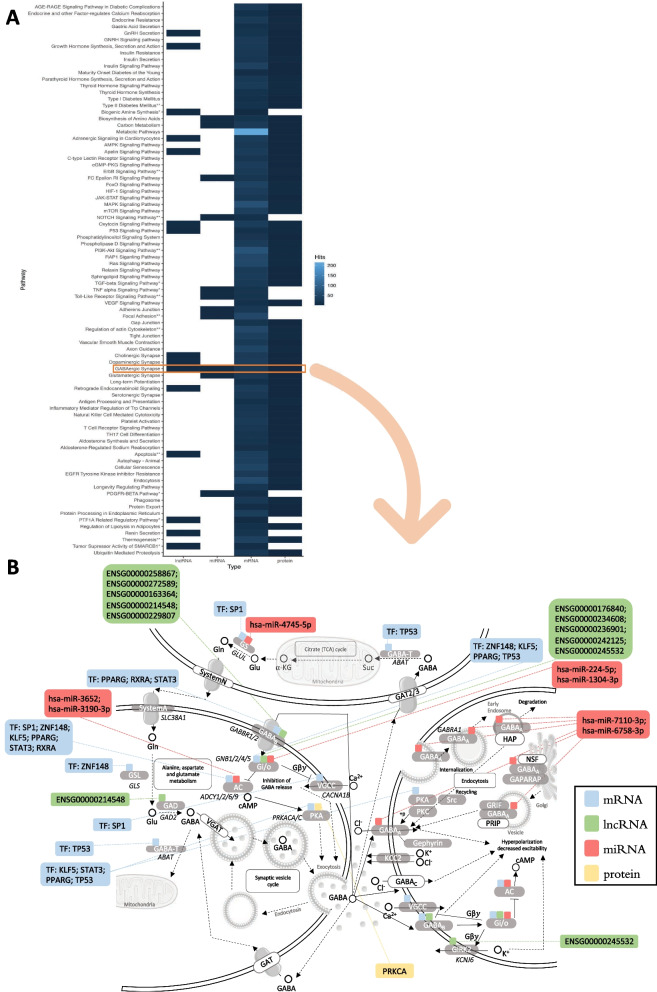
Integrated functional analysis. **A** Heatmap showing the main enriched pathways common to the differentially expressed components messenger RNA (mRNA), long noncoding RNA (lncRNA), microRNA (miRNA), and proteins. The colors represent the number of hits for each type. **B** GABAergic synapse functional pathway interactions and regulation of altered factors. The transcription factors (TFs) regulating differentially expressed mRNA genes are represented in blue. Modulations by lncRNAs and miRNAs are represented in green and red, respectively. The protein identified is in yellow

The key players for each RNA type (mRNA, lncRNA, and miRNA) and associated proteins were integrated into a functional analysis to uncover potential diabetes markers. As expected, pathways associated with T1D, T2D and maturity-onset diabetes of the young appeared altered in mRNA and associated proteins. Insulin signaling, secretion, and resistance were also altered. Several signaling pathways (*e.g.*, MAPK, FoxO, PI3K-Akt) were altered. Retrograde endocannabinoids, growth hormone processing and secretion, glutamatergic, dopaminergic and cholinergic synapses, carbon metabolism and amino acid synthesis, and apoptosis were some of the pathways common to three groups. The enriched pathway analysis of DEGs, proteins, and targets of miRNAs and lncRNAs showed a common pathway to all players, the GABAergic synapse (Fig. 6A). This pathway is regulated at different levels, schematically represented in Fig. 6B, in which intervening components of the pathway are regulated by several TFs, miRNAs, and lncRNAs shown to be differentially expressed in DD.

### Comparative analysis with genome-wide association studies

**Fig. 7:**
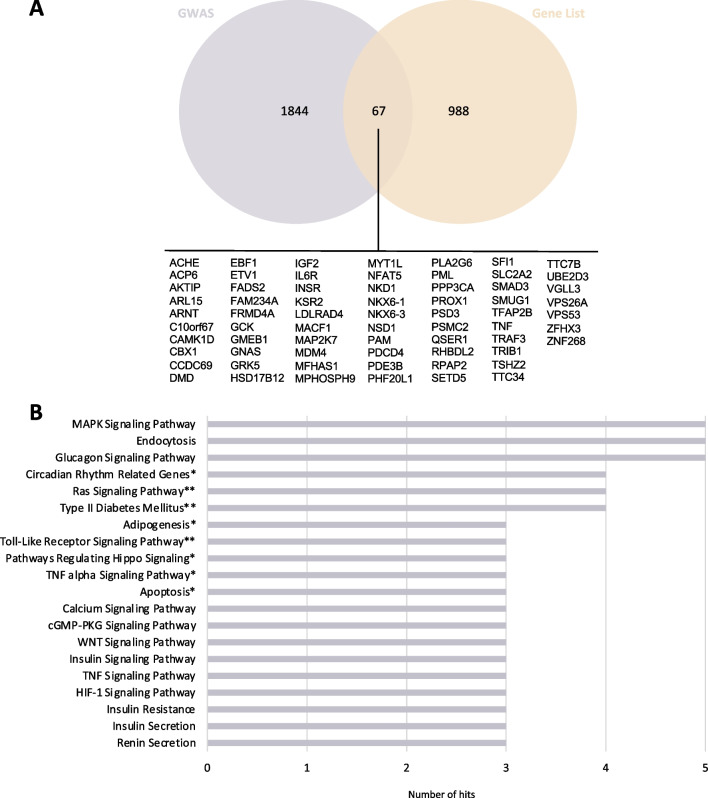
Integrative analysis with genome-wide association studies (GWAS). **A** List of genes common between the GWAS Catalog for diabetes and the list of differentially expressed genes (DEGs) common for mRNA, lncRNA and miRNA obtained in this study. **B** Histogram of the top 20 altered pathways. KEGG pathways are represented with no asterisk, WikiPathways with *, and pathways common to both databases are represented with **

The association of diabetes-modulated genes from the GWAS catalog and the DEGs obtained in this study revealed 67 common genes (Fig. 7A). To gain further insight into the effects of diabetes, we performed a functional enrichment analysis of these common genes using GeneTrail. The top 20 pathways with a higher number of hits were associated with T2D, glucagon, insulin, MAPK, and Ras signaling and endocytosis, among others (Fig. 7B).

## Discussion

Diabetes pathogenesis is not yet fully understood due to the heterogeneity and complex underlying disease mechanisms. The loss of pancreatic β-cell mass, by either autoimmune destruction or apoptosis, represents a pathophysiological process leading to insulin deficiency in diabetes. Therapeutic strategies focusing on restoring β-cell mass and β-cell insulin secretory capacity may impact disease management. Thus, this study aimed to investigate diabetes-related gene expression studies to uncover molecular targets and networks associated with diabetes.

Over the years, transcriptomic studies have shed light on common molecular mechanisms underlying T1D and T2D and the early molecular changes associated with insulin resistance and impaired fasting glucose [35]. Nevertheless, while individual analysis pinpoints relevant mechanisms, the integrated and combined analysis of multiple levels emerges as a promising approach to comprehending disease-linked mechanisms. Integrated strategies have the power to expand our knowledge of disease-related determinants that can be used as biomarkers of disease onset and progression [36]. Despite the massive data provided by different studies, limitations and challenges still affect the results, from the choice of materials to the methods applied [35].

In this study, several diabetes-associated components were identified through the analysis and integration of gene expression, lncRNAs, and miRNAs data. As predicted, the highest percentage of significantly altered genes corresponded to protein-coding genes. Most of these genes were upregulated, as is generally found in most studies [15, 37]. These genes are typically involved in pathways such as cell signaling, the immune system, cell adhesion and interactions, metabolism, and the nervous system, among other cellular functions, revealing dysregulated activation of several β-cell mechanisms.

Metabolic pathways, although not common to all studies, have the highest number of hits and altered genes. Many of these altered genes are mainly involved in mitochondrial function and oxidative phosphorylation, carbon metabolism, and amino acid synthesis. Several studies have highlighted the association between mitochondrial dysfunction and β-cell dysfunction. Moreover, mitochondrial dysfunction in many tissues can contribute to diabetes pathogenesis and complications in several ways [38]. An increase in oxidative stress due to mitochondrial dysfunction can cause functional alterations in proteins, lipids and nucleic acids, and interfere with different cellular processes [39]. Additionally, mitochondrial metabolism is crucial for the coupling of amino acids that, under appropriate conditions, can stimulate insulin secretion. In fasting periods, a few amino acids may modulate glucagon release from pancreatic α-cells, which then influences insulin secretion from β-cells [40]. Further supporting the link between mitochondrial function and amino acid metabolism, this study also identified several genes associated with amino acid synthesis, such as *ALDOB*, *ARG2*, *ENO1*, and *GLU*, which are differentially expressed under diabetic conditions.

Olfactory transduction appears to be a common pathway in all studies. Changes in olfactory sensitivity and loss of olfactory function were reported both in T1D and T2D [41]. Moreover, it was shown that olfactory impairment is a predictive clinical sign of neurological defects in elderly people with T2D. Hence, olfactory alterations could be used as an early biomarker to detect incoming cognitive problems in the context of metabolic diseases [41]. An increasing number of olfactory receptors that mediate chemo-sensing in olfactory transduction in neurons have been identified in insulin-producing pancreatic islets, mainly in α- and β-cells. Previous studies have shown that olfactory receptors in pancreatic α- and β-cells can modulate glucagon and insulin secretion, respectively, in a cell-autonomous manner [42]. This system can modulate glucose metabolism and be a potential therapeutic target for diabetes by enhancing insulin secretion [42].

Interestingly, the FoxO signaling pathway was also altered in 4 of the 6 studies selected for the analysis. FoxO proteins are transcription factors involved in numerous physiological processes and various pathological conditions, including diabetes. In particular, FoxO1 is an important regulator of pancreatic β-cell function, improving cell compensation under metabolic stress, and it also regulates α-cell mass by controlling Arx expression [43, 44]. Furthermore, the loss of FoxO1 signaling in response to metabolic stress promotes the dedifferentiation of β-cells to a cell type similar to endocrine progenitors. Additionally, studies have identified dedifferentiated β-cells in human islets from diabetic donors and showed that these cells can start expressing glucagon, becoming functionally similar to α-cells [44].

Regarding the pathways encountered in the different original publications, several pathways are common. Pathways associated to metabolism (mainly glucogenesis), cell signaling and diabetes (T2D and maturity-onset diabetes of the young (MODY)) are present in most of the studies. Moreover, alterations associated with insulin signaling were found in GSE86473 and GSE76894. Additionally, changes in cell adhesion, immune response, apoptosis, mitochondrial function, and proteosome, were suggested in GSE86473, GSE164416, GSE20966 and GSE124742. Also, Wnt and Il-1 signaling pathways were described in GSE76894 and GSE124742. Furthermore, GSE124742 points to alterations on the regulation of β-cell development, ion channel transport, notch signaling, regulation of gene expression in β-cells. Interestingly, GSE76894 refers alterations in GABA receptor signaling pathway.

However, the differences previously reported for gene expression between the studies are also reflected on pathway analysis. Apart from the differences intrinsic to the different samples, the diversity of statistical methods (over-representation analysis vs. gene set enrichment analysis and different *p* value adjustment methods) and database resources (KEGG, WikiPathways, Gene ontology) applied could also account for the differences found.

The expression of genes and proteins can also be regulated post-transcriptionally by noncoding RNAs, such as lncRNAs and miRNAs. Both lncRNAs and miRNAs have been shown to execute vital roles in the regulation of diabetes pathophysiological processes [7].

Recent studies have shown changes in lncRNA expression in individuals with diabetes or animal models of the disease. This suggests that lncRNAs may play important roles in the regulation of β-cell function, impacting insulin secretion and glucose metabolism, which are often dysregulated during pancreatic β-cell differentiation [8]. A fraction of β-cell lncRNAs are cell-specific and are activated during β-cell differentiation. This lncRNA’s cellular specificity has also been reported in other cell types, suggesting a role for lncRNAs in the regulation of lineage-specific differentiation or specialized cellular functions [45]. They can also contribute to diabetes complications by altering mechanisms involved in inflammation, endoplasmic reticulum (ER) stress, and mitochondrial dysfunction [46].

For the differentially expressed lncRNAs identified, *MIR7-3HG, LINC01116, HIF1A, MEG3, XIST, NEAT1*, and *HHLA3* have been previously associated with some form of diabetes and diabetes-associated complications [8, 47–52]. *MEG3* is downregulated in the islets and blood of individuals with diabetes, and in a murine β-cell line (MIN6), *MEG3* seems to regulate insulin synthesis and secretion since its absence reduced the expression of key factors for β-cell function (*PDX-1* and *MAFA*), decreasing insulin synthesis [8, 52]. *NEAT1* is overexpressed in individuals, and it is highly expressed in hyperglycemia conditions affecting inflammatory pathways [53, 54]. *MIR7-3HG* was shown to ameliorate dexamethasone-induced dysfunction in β-cells through the PI3K-AKT signaling pathway [47].

Regarding the altered lncRNA-associated pathways, the results are in accordance with those of the protein-coding genes in the sense that metabolic pathways have the highest number of genes involved, and olfactory transduction is altered in almost all studies. Additionally, the pathways of lysine degradation and circadian entrainment were also altered in all studies. Similarly, these pathways were enriched in the miRNA analysis along with pathways associated with inflammation, cellular signaling, and apoptosis, as will be discussed next.

MiRNAs are highly stable and can be found in several tissues and body fluids, including human peripheral blood, suggesting that they can be a source of accessible biomarkers of the disease [7]. Over the years, an increasing number of miRNAs have been associated with diabetes pathogenesis. Dysregulation of miRNAs can lead to profound impairment of glucose metabolism since they can affect β-cells through different mechanisms, such as apoptosis, proliferation, differentiation, or altered function, particularly insulin secretion [55]. In pancreatic islets, most studies this far have investigated the role of single miRNAs and suggest that some miRNAs can exert compensatory effects on β-cells and others impact insulin secretion through miRNA-mediated dysfunction. However, it is suggested that islet function is regulated by miRNA groups rather than single miRNAs. A regulatory network resorting to whole-genome data and bioinformatic tools to scrutinize diabetes-related differentially expressed miRNAs and their targets can provide more accurate information about the pathophysiology of the disease [56]. Among the specific islet miRNAs found to be altered in our study, miR-24-3p, miR7110-3p, miR3652, miR-4745-5p, and miR224-5p were increased in diabetes conditions [57–[62]. For example, miR7110 was present in individuals with T1D, and *IGF2BP2* (insulin-like growth factor 2 mRNA binding protein 2) is a direct target of this miRNA [58]. Furthermore, miR-24-3p interferes with the PI3K/AKT pathway, which has an important role in cell proliferation and differentiation [63]. Nevertheless, studies about the specific mechanisms and targets through which miRNAs function in diabetes and their impact on β-cell dysfunction are still scarce [64].

Furthermore, transcription factors can be responsible for the regulation of the DEGs. In this study, we identified 16 potential TFs that could regulate some of the altered genes. Among these, HIPK2 activity has been found to be affected by hyperglycemia conditions and also influences the activity of PDX-1 [65, 66]. Additionally, KLF5 interacts with FOXO1 and is suggested to be involved in T1D and T2D, and in diabetic complications [67, 68]. PPARG and RXR have been suggested as therapeutic targets for T2D, due to PPARG role in regulating genes associated to glucose homeostasis [69], and RXR for regulating glucose-stimulated insulin secretion and affecting a number of genes that have been implicated in β-cell function and differentiation [70]. STAT1 mediates β-cell dedifferentiation, inflammation and apoptosis pathways [71]. Lastly, STAT3 expression has been associated with β-cell dysfunction and promotion of neonatal diabetes [72]. Moreover, STAT3 has been suggested to play a role in regulating cellular plasticity and in α- to β-cell transdifferentiation [73].

The integrated analysis showed several altered pathways common to at least 3 groups. For example, the endocannabinoid pathway can contribute to β-cell loss by modulating inflammatory and cell death mechanisms, and dysregulation in this system can have deleterious effects on glucose metabolism and insulin sensitivity [74]. The prevalence of different altered synapses, such as glutamatergic, dopaminergic and cholinergic synapses, agrees with recent studies that show impairment in synaptic processes as a result of defects in insulin action, suggesting that neurotransmitter systems are susceptible to insulin signaling abnormalities [75]. Moreover, other signaling pathways (*e.g.*, MAPK, FoxO, and PI3K-Akt) were also identified. PI3K-Akt signaling is vital for insulin function, and in diabetes, impairments in this pathway can lead to insulin resistance by inducing oxidative stress, protein accumulation and misfolding, mitochondrial dysfunction, inflammation, and apoptosis [76].

GABAergic synapses emerge as a common pathway for all players. γ-Aminobutyric acid (GABA) is an important neurotransmitter that is highly produced in the central nervous system and is also expressed in pancreatic β-cells. In pancreatic islets, GABA is associated with auto- and paracrine signaling between endocrine cells. Several studies have shown that this neurotransmitter is involved in glucose-responsive insulin and negatively regulates glucagon release. Dysregulation of α-cell glucagon secretion contributes to hyperglycemia present in T1D and T2D. Furthermore, GABA content is reported to be reduced in human islets from T1D and T2D donors, and the functional consequences of this process have not been studied [77]. Remarkably, this study reveals that several players in this system, including glutamic acid decarboxylase (GAD) and GABA_A_ receptor, which are essential for GABA metabolism, are regulated by miRNAs and lncRNAs that are altered in donors with diabetes. For example, miR7110-3p, miR3652, miR-4745-5p, and miR224-5p have been identified in the GABAergic pathway, in which miR7110-3p is involved in the regulation of the GABA_A_ receptor. This receptor has been identified in β-cells, and its expression and response have been found altered in human islets from donors with T2D [77]. Furthermore, lncRNAs *MIR7-3HG, LINC01116, HIF1A, MEG3, XIST* and *NEAT1* have also been identified in the GABA system, in which *MEG3* is involved in the regulation of GAD. GAD is responsible for the synthesis of GABA in β-cells and is also a major autoantigen in T1D [77].

Additionally, exogenously delivered GABA has been reported to improve β-cell mass in conditions of β-cell depletion by promoting transdifferentiation of α-cells into β-cells in insulin-deficient or diabetic mice [78]. The potential for GABA to effectively influence β-cell mass in vivo and the functional consequences of its reduction in donors with diabetes has not been thoroughly demonstrated. Remarkably, GABA appears to have an important role in β- and α-cell communication [77], suggesting that the players in this islet GABAergic system can be potential targets for novel diabetes therapeutic strategies with a particular focus on cell transdifferentiation.

Diabetes-associated variants, mostly located in noncoding regions, are associated with β-cell function and mechanisms. However, the association between the identified risk loci and mechanisms underlying disease onset and progression is still difficult [15, 56]. Combining the integrative analysis with GWAS data empowered the relevance of alterations in disease conditions and highlighted several pathways. For instance, glucagon signaling has an essential role in intra-islet paracrine regulation and insulin secretion, reinforcing the importance of communication between α- and β-cells.

Despite the relevance of the results presented here, they should be interpreted with caution considering the study’s limitations. First, the variability of individual donors in terms of their underlying pathophysiological condition must contribute to the evident differences between the expression profiles of individuals with diabetes and those without the disease so that the results presented here need to be experimentally validated. In addition, limitations associated with the variability between studies, namely the experimental protocols (*e.g.,* differences in islet isolation and culturing techniques, the number of samples under comparison) can create a bias in the results towards the studies with a higher number of DEGs. Furthermore, the differences in the original analysis (*e.g.,* distinct profiling methods) may also influence the obtained results.

## Conclusions

This study took advantage of powerful bioinformatic tools to scrutinize and integrate data from publicly available diabetes-associated gene expression data, highlighting molecular targets associated with β-cell dysfunction. The integrated analysis of protein-coding genes, miRNAs and lncRNAs identified several pathways involved in important cellular functions, such as metabolism, cell signaling, the immune system, cell adhesion and interactions. The GABAergic synapse pathway was the common pathway to all datasets analyzed. Considering the importance of GABA in pancreatic islets, this process could be a potential therapeutic target through the transdifferentiation of α-cells into β-cells.

### Supplementary Information

**Additional file 1.** List of genes for the differentially expressed genes (DEGs) for each study analysed (GSE164416, GSE25724, GSE76894, GSE20966, GSE86743, GSE124742).

**Additional file 2.** Network of potential transcription factors (TFs) and their targets. The common TFs to at least 3 studies are represented as nodes and their targets as the edges.

## Data Availability

The raw datasets supporting the conclusions of this article will be made available by the authors, without undue reservation, to any qualified researcher. The original data from each study analyzed here are publicly available. The data can be found here: https://www.ncbi.nlm.nih.gov/geo/query/acc.cgi?acc=GSE164416, https://www.ncbi.nlm.nih.gov/geo/query/acc.cgi?acc=GSE25724, https://www.ncbi.nlm.nih.gov/geo/query/acc.cgi?acc=GSE76894, https://www.ncbi.nlm.nih.gov/geo/query/acc.cgi?acc=GSE86473, https://www.ncbi.nlm.nih.gov/geo/query/acc.cgi?acc=GSE124742, https://www.ncbi.nlm.nih.gov/geo/query/acc.cgi?acc=GSE20966.

## Abbreviations

BMI: Body mass index
DD: Donors with diabetes
DEG: Differentially expressed genes
ER: Endoplasmic reticulum
FACS: Fluorescence-activating cell sorting
GABA: γ-Aminobutyric acid
GAD: Glutamic acid decarboxylase
GEO: Gene Expression Omnibus
GWAS: Genome-Wide Association Studies
IDF: International Diabetes Federation
KEGG: Kyoto Encyclopedia of Genes and Genomes
lncRNA: Long noncoding RNA
miRNA: MicroRNA
MODY: Maturity-onset diabetes of the young
mRNA: Messenger RNA
ncRNA: Noncoding RNA
ND: Donors without diabetes
T1D: Type 1 diabetes
T2D: Type 2 diabetes
TF: Transcription factor
