# Supplementary figures and images for: Gene expression analysis reveals diabetes-related gene signatures
Source: Hum Genomics. 2024 Feb 8;18:16. doi: 10.1186/s40246-024-00582-z (PMC10851551; doi:10.1186/s40246-024-00582-z)

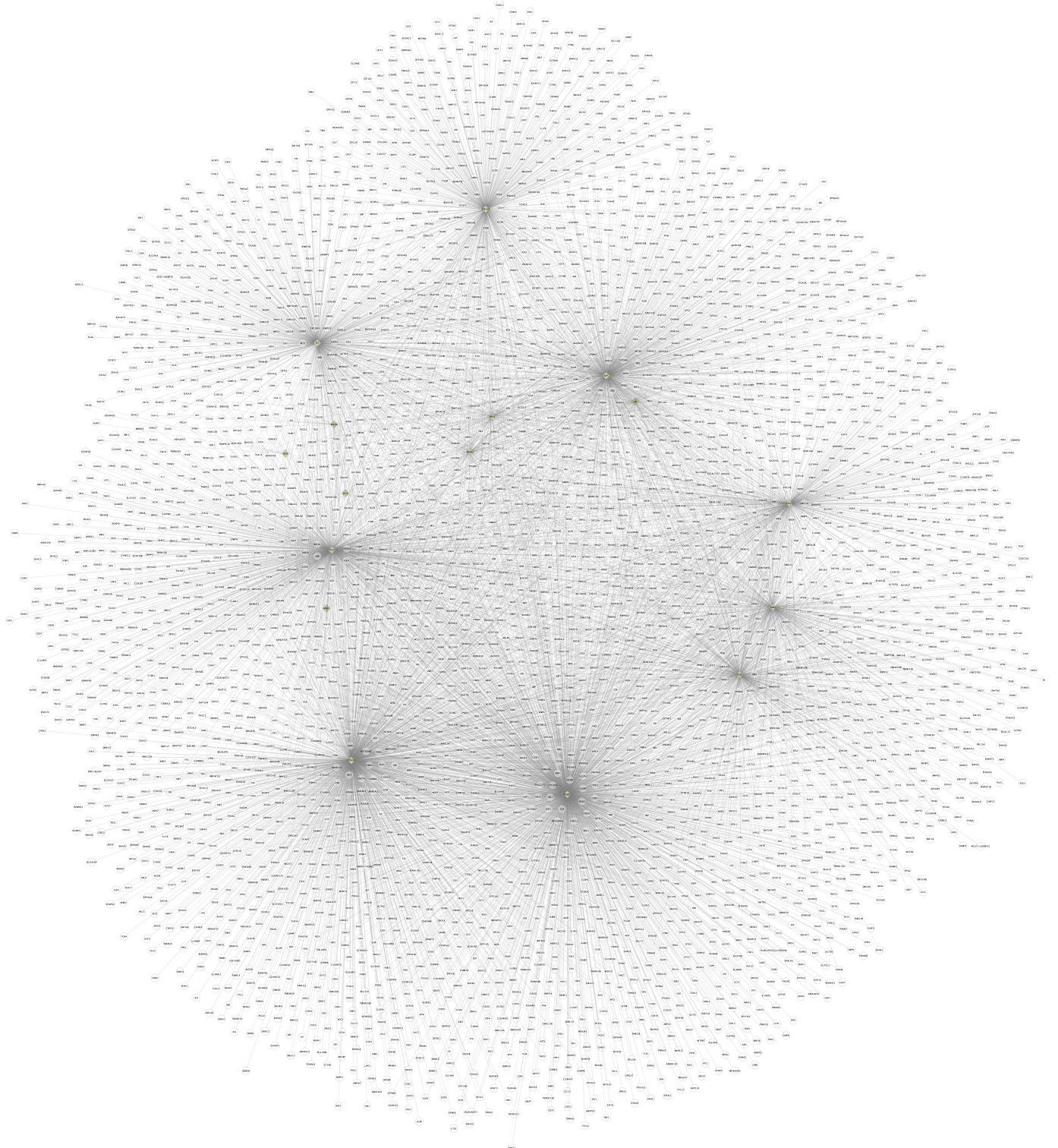

Supplement: Supplementary file 2 — Additional file 2. Network of potential transcription factors (TFs) and their targets. The common TFs to at least 3 studies are represented as nodes and their targets as the edges. [file 40246_2024_582_MOESM2_ESM.pdf]
